# COMPARING THE FEASIBILITY OF A LONELINESS AND SOCIAL ISOLATION GROUP IN A PRIMARY CARE VS COMMUNITY SETTING

**DOI:** 10.1093/geroni/igad104.3239

**Published:** 2023-12-21

**Authors:** Max Zubatsky, Marla Berg-Weger, Dimitra Tziarli, Rachel Livingston, Judy Drake, Ashley Taylor

**Affiliations:** Saint Louis University, St. Louis, Missouri, United States; Saint Louis University, St. Louis, Missouri, United States; Saint Louis University, St. Louis, Missouri, United States; Saint Louis University, St. Louis, Missouri, United States; Family Care Health Centers, St. Louis, Missouri, United States; Saint Louis University, St. Louis, Missouri, United States

## Abstract

Social isolation and loneliness is now considered a public health epidemic in the United States for the older adult population. While providers try to address certain health and lifstyle issues in this population through medications and other medical referrals, there are few non-pharmacological group interventions to curb feelings of isolation and loneliness. This study will determine if a psychosocial group intervention in primary care results in decreases of loneliness and health risks compared to a community-based group. The intervention is called “Circle of Friends,” which is an evidence-based group to alleviate loneliness and increase socialization through a rehabiliation model of care. Participants must be 60 years and older, report subjective feelings of loneliness, have reported being homebound and/or isolated over the last six months, and have very few social connections. The current study has collected initial pre-group data from eight older adults from the community group, and the research team is recruiting participants in the primary care group setting. It is anticipated that the Primary Care Circle of Friends Group will report higher improvements in loneliness and health indicators compared to the community-based group. The primary group will take place in an underserved Federally Qualified Health Center, and the community-based group will take place in the conference room of a university setting. Early qualitative themes that facilitators have reported around recruitment include: 1.) Challenges with planning transportation, 2.) Building trust in joining a new group, and 3.) requests of extra resources in addition to this group.

